# Wildcard Fields-Based Partitioning for Fast and Scalable Packet Classification in Vehicle-to-Everything

**DOI:** 10.3390/s19112563

**Published:** 2019-06-05

**Authors:** Jaehyung Wee, Jin-Ghoo Choi, Wooguil Pak

**Affiliations:** 1 Computer Engineering Department, Keimyung University, Daegu 42601, Korea; wee2016@kmu.ac.kr; 2 Department of Information and Communication Engineering, Yeungnam University, Gyeongsan 38541, Korea; jchoi@yu.ac.kr

**Keywords:** network security, packet classification, high-speed communication, high scalability

## Abstract

Vehicle-to-Everything (V2X) requires high-speed communication and high-level security. However, as the number of connected devices increases exponentially, communication networks are suffering from huge traffic and various security issues. It is well known that performance and security of network equipment significantly depends on the packet classification algorithm because it is one of the most fundamental packet processing functions. Thus, the algorithm should run fast even with the huge set of packet processing rules. Unfortunately, previous packet classification algorithms have focused on the processing speed only, failing to be scalable with the rule-set size. In this paper, we propose a new packet classification approach balancing classification speed and scalability. It can be applied to most decision tree-based packet classification algorithms such as HyperCuts and EffiCuts. It determines partitioning fields considering the rule duplication explicitly, which makes the algorithm memory-effective. In addition, the proposed approach reduces the decision tree size substantially with the minimal sacrifice of classification performance. As a result, we can attain high-speed packet classification and scalability simultaneously, which is very essential for latest services such as V2X and Internet-of-Things (IoT).

## 1. Introduction

Internet traffic is increasing exponentially every year with the advent of new services such as Internet-of-Things (IoT) and Vehicle-to-Everything (V2X). The number of connected vehicles is expected to reach 125 million by 2022 [1]. In addition, the number of IoT devices is increasing over 26 billion, so we expect the traffic will soar up even faster in the near future [2]. With more traffic, security rules installed on the network equipment also grow in size and complexity. When we apply the security rules to incoming packets, we use an algorithm called packet classification, which requires a large searching table and much processing time. 

The packet classification algorithm is not only necessary for security function, but also essential for basic packet processing in network equipment [3]. For the operation of the algorithm, we first define the rule-set specifying how to handle the received packets. The rule-set is a large collection of rules, and each rule consists of a priority and matching conditions for each field in the packet header. Whenever a packet arrives, the algorithm searches for matching rules by comparing each rule with the fields in the header, and chooses the highest priority rule among them. Hence, in order to handle vast traffic, it is critical for network equipment to perform packet classification fast and efficiently. In other words, the packet classification algorithm is one of the key components that determines the performance of network equipment. Recently, networks are growing in size and complexity tremendously, and the rule-set size is increasing in proportion. Therefore, the packet classification algorithm should be capable of processing packets at very high speed, even with the huge rule-set.

To resolve this issue, various packet classification algorithms have been proposed in previous literature [4,5,6]. The algorithms can be categorized into software and hardware-based approaches. Hardware based algorithms need specialized hardware chips to boost the classification performance. However, they cannot support high flexibility to customers’ various demands [7,8,9]. For software-based algorithms, some algorithms build a small search table to accommodate a large rule-set with limited memory [10,11]. Others focus on speeding up the classification process by spending more resources, in order to support vast traffic [12]. We also propose an approach to increase search speed especially for multi-classification required [13].

Unfortunately, there is a common tradeoff between search table size and packet classification speed. For example, our approach can improve the classification speed significantly but also suffers from a huge table size. Thus, when designing a packet classification algorithm, a popular guideline is to improve the classification speed by using more memory. Most existing algorithms are based on such an approach and do not satisfy high classification speed and small memory usage at the same time.

Today a decision tree, which is a well-known searching algorithm, is widely used in packet classification algorithms. The algorithms construct a decision tree by dividing a rule-set into multiple sub-partitions repeatedly, according to an internal criterion that determines the partitioning fields and the partitioning size. When a packet arrives, the algorithms search through the decision tree, and find a matching rule. Unfortunately, although the decision tree is very popular, we cannot construct the optimal decision tree in the computationally efficient manner. Indeed, even the two-means clustering, i.e., the simplest partitioning problem of dividing a set into two partitions is NP-hard [14,15]. Many decision tree-based packet classification algorithms have been developed and adopted in the commercial equipment, but they build just a suboptimal decision trees due to the computational complexity.

In packet classification algorithms using the decision tree, rules in a node are partitioned into multiple subsets of the rules and each subset is stored in a child node. If a rule satisfies conditions of more than one partition, it is stored in multiple child nodes. In that case, we say the rule is duplicated. If the rule duplication happens frequently, we consume more memory space for the decision tree because the same rule is copied several times in the memory. Hence, we should be careful to choose a partitioning criterion in order to minimize the rule duplication. 

In this paper, we propose a new partitioning algorithm for decision tree-based classification algorithm to minimize rule duplication significantly. To this end, we define a partitioning preference coefficient for each partitioning field. The value represents how uniformly rules can be distributed in the node and how efficiently the rule duplication can be avoided, when each field is chosen as the partitioning criterion. We define the coefficient as simply as possible since the computational burden can degrade the partitioning performance. This algorithm can be applied for most decision tree-based packet classification algorithms and can greatly improve their scalability without sacrificing classification performance. Since our algorithm improves the common partitioning procedure of decision tree-based algorithms, it can be applied for most decision tree-based algorithms without significant modification.

Our contribution is twofold as follows:

- First, we propose a new partitioning field selection algorithm that finds the optimal partitioning field using the number of unique matching ranges and the number of wildcards. As far as we know, it is the first approach to use the concept of wildcards based on the matching range of each node, so the algorithm can minimize duplicated rules compared to existing ones.
- Second, we also propose a new partitioning number per field decision algorithm that chooses two partitioning fields through the partitioning field selection algorithm on each node, and finds the number of partitions based on the selected fields to minimize rule duplication. Since it considers only two fields to choose the partitioning number in contrast to existing algorithms using multiple fields, it is fast without the performance degradation.

We organize the remainder of this paper as follows. We briefly explain the related works in Section 2, and describe the motivation, detailed operation, and features of the proposed algorithm in Section 3. In Section 4, we analyze the performance of existing algorithms before and after adopting our proposed algorithm. Conclusion follows in Section 5.

## 2. Related Works

In this section, we explain two famous packet classification algorithms based on the decision tree, i.e., HyperCuts [12] and EffiCuts [16]. HyperCuts attains high classification speed while requiring a huge table. On the contrary, EffiCuts shows much smaller table size but at a sacrifice of classification performance.

### 2.1. HyperCuts

HyperCuts extends the previous HiCuts algorithm [17]. However, HyperCuts has its own unique features. That is, when generating a decision tree, HyperCuts performs the equal-interval partitioning based on multiple fields, while its predecessor, HiCuts considers only a single field. This multi-field-based partitioning greatly improves packet classification performance and memory requirements simultaneously.

Before describing HyperCuts in detail, we explain the meaning of “the number of unique matching ranges”. We consider the rule-set in Table 1 as an example. Each rule consists of four fields, and each field has a range of matching values. One distinct range of matching values in each field is called “unique matching range”. For example, each rule has one matching range for Field 1 in Table 1, so the set of unique matching ranges is {(0,8), (6,9), (0,15), (9,10), (6,15), (0,2)}. Here, the number of unique matching ranges is 6.

When generating a decision tree, HyperCuts needs two configuration parameters, “binth” and “space factor”, where “binth” is the maximum number of rules stored in the leaf node and “space factor” is the maximum number of rule duplications allowed in each partitioning operation. To satisfy the constraints, HyperCuts builds the decision tree as follows. At first, it partitions a root node that contains the entire rule-set, based on one or more fields. As a result, we obtain multiple subsets of rules, and to store each subset, corresponding child nodes are generated. This procedure repeats until all the leaf nodes satisfy the constraints. During the procedure, HyperCuts selects the partitioning fields and the number of partitions for each field. We describe each step in detail. 

#### 2.1.1. Partitioning Field Selection

HyperCuts should choose the effective partitioning fields prior to conducting partitioning. However, it is not easy to select optimal partitioning fields. When the number of partitioning fields is large, more subsets (and accordingly more child nodes) are generated. Hence, the height of the decision tree decreases, and thus, classification performance improves. Instead, memory usage increases since each rule can be duplicated to multiple child nodes. We should choose an appropriate number of partitioning fields to balance search performance and memory requirement simultaneously. For this, HyperCuts selects one or more fields for partitioning, considering the number of unique matching ranges. For illustration, we define the following notations.

- d: Total number of fields in rules.
- $u_{k}$: Number of unique matching ranges for the *k*-th field, where k∈{1,2,⋯,d}.
- $\bar{u}=\frac{1}{d}\sum_{k=1}^{d}u_{k}$: Average number of unique matching ranges.

In HyperCuts, all fields satisfying Equation (1) are chosen as partitioning fields:

(1) $$u_{k}\geq \bar{u}\;\mathrm{and}\;u_{k}\neq 1$$

For example, if we count unique matching ranges for each field in Table 1, $u_{k}$’s are 6, 6, 3, and 2 for k = 1, 2, 3, and 4, respectively. Their average is 4 and according to Equation (1), Fields 1 and 2 become the partitioning fields.

#### 2.1.2. Partitioning Number Decision

After choosing partitioning fields, HyperCuts determines how many child nodes it should generate by partitioning the current node. The number of partitions affects memory requirement as well as classification speed. Unfortunately, it is inefficient and almost impossible to look over all the possible combinations of fields. Alternatively, HyperCuts calculates the number of near-optimal partitions, considering each field one-by-one. To describe the approach, we define the following symbols.

- n: Total number of rules in the current node.
- c(k): Number of partitions for the k-th field.
- ρ: Space factor.
- $N_{max}$: Maximum number of partitions.

Now, the maximum c(k) satisfying Equation (2) is determined as the number of partitions for the k-th field.

(2) c(k)<ρ·n

Since HyperCuts conducts partitioning for multiple fields, the total number of partitions is $c\left(1\right)c\left(2\right)\cdots c\left(d\right)=\prod_{k=1}^{d}c\left(k\right)$. Thus, HyperCuts creates many child nodes and consumes a large amount of memory. In order to resolve this issue, the algorithm repeatedly reduces the number of partitions by half, until it satisfies Equation (3), for the field whose number of partitions is the smallest. This operation is beneficial to avoid excessive memory usage.

(3) $$\overset{d}{\underset{k=1}{\prod }}c\left(k\right)<\min\left(\rho \cdot \sqrt{n},\;N_{\max}\right)$$

**Figure 1:**
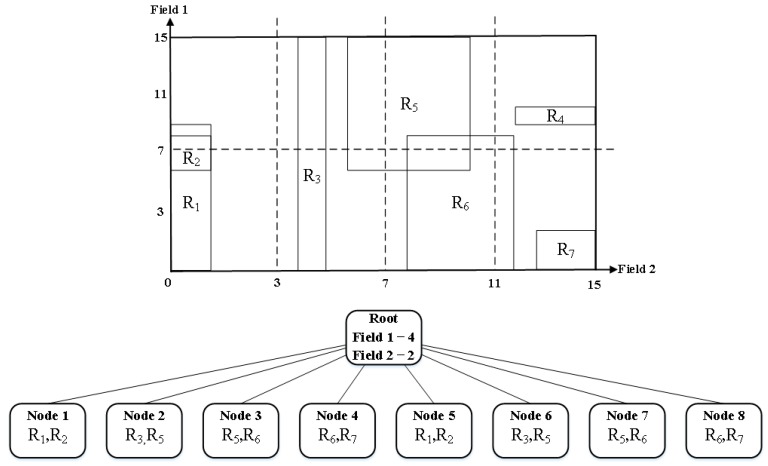
Decision tree generated from Table 1 by HyperCuts, where both binth and space factor are set to 2 both.

When more than one field has the same partition number, the algorithm prefers the field with the smallest matching range. For the rule-set of Table 1, HyperCuts constructs a decision tree of Figure 1, by choosing the partitioning fields, deciding the number of partitions for each field, and recursively conducting the partitioning for each node with more rules than “binth”.

### 2.2. EffiCuts

EffiCuts aims at cutting the memory requirement down because it is a critical problem of HyperCuts in practice. EffiCuts builds a decision tree based on HyperCuts. However, differently from HyperCuts, it creates multiple decision trees for each subset of rules, called “category”. This approach reduces the rule duplication significantly.

We now explain two important operations of EffiCuts, i.e., tree splitting and tree merging. We here assume that each rule consists of 5-tuple of source IP, destination IP, source port, destination port, and protocol.

#### 2.2.1. Tree Splitting 

EffiCuts constructs multiple decision trees, contrary to HyperCuts. To this end, the algorithm splits the entire rule-set into categories in advance, where a “category” means a group of rules with similar matching ranges for each field. Specifically, EffiCuts defines the wildcard field for each rule using the following notations:

- $FK_{\min}^{k}$: Minimum value matching the k-th field. e.g., 0 for protocol field.
- $FK_{\max}^{k}$: Maximum value matching the k-th field. e.g., 255 for protocol field.
- $PK_{\min}^{k}$: Minimum value matching the k-th field of a given rule.
- $PK_{\max}^{k}$: Maximum value matching the k-th field of a given rule.

That is, the k-th field is a wildcard of a rule if it satisfies Equation (4).

(4) $$\left(PK_{\max}^{k}-PK_{\min}^{k}\right)\geq \left(FK_{\max}^{k}-FK_{\min}^{k}\right)\cdot \Delta$$

where Δ is the minimum wildcard ratio, usually set to 0.5. If a field of any rule is a wildcard, we call the field as ‘wildcard field’. We explain the concept of wildcard using the example of Table 1. As the size of Field 1 is 4-bit, it becomes a wildcard field if there exists any rule whose matching range for Field 1 is larger than or equal to (15−0)×0.5=7.5, according to Equation (4). Thus, Field 1 becomes a wildcard field for Rule 3, 5, and 6. We determine whether each field is wildcard field or not, and classify all the rules into four categories according to the number of wildcard fields.

- Category 1: rules with four wildcard fields
- Category 2: rules with three wildcard fields
- Category 3: rules with two wildcard fields
- Category 4: rules with one or zero wildcard field

When each rule consists of 5-tuple, we can divide Category 1 further into five subcategories; all fields are wildcard except for source IP, or destination IP, or source port, or destination port, or protocol. Similarly, we can define ten subcategories for Categories 2 and 3 and six subcategories for Category 4. Conclusively, we classify the rules of Table 1 into 31 subcategories. EffiCuts creates a decision tree for each subcategory because rule duplication decreases significantly when the rules have similar characteristics in all fields.

#### 2.2.2. Tree Merging

**Figure 2:**
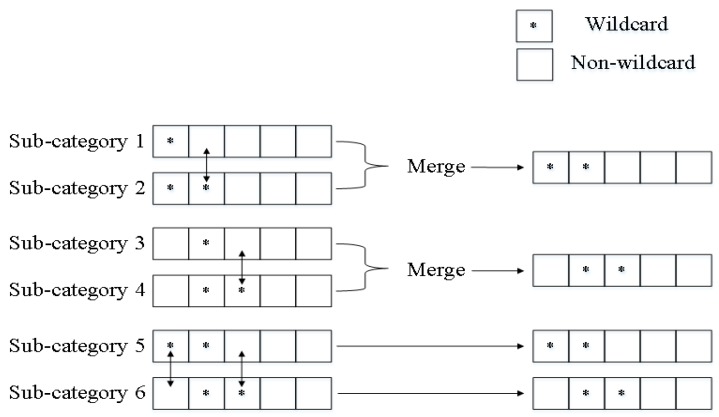
Example of merging subcategories to reduce the number of decision trees in EffiCuts.

If we create decision trees for each subcategory using EffiCuts, memory usage decreases drastically since rule duplication is minimized. However, we should look up all the decision trees for classification of each packet, which deteriorates the search performance. Thus, we need to limit the total size of decision trees within a certain number. In EffiCuts, we can reduce the number of decision trees by merging multiple subcategories into one. Unfortunately, the merge can lead to rule duplication. To minimize this negative effect, EffiCuts combines similar subcategories only, specifically the subcategories different in one wildcard field at most. For example, in Figure 2, subcategories 1, 2, 3, and 4 have one different wildcard field each other. Therefore, any two subcategories among them can be merged into a single subcategory. On the other hand, subcategories 5 and 6 cannot be merged because they have two different wildcard fields. Tree merging process repeats by comparing all subcategories, selecting two subcategories, merging them into one category, and constructing a decision tree in the same way with HyperCuts.

EffiCuts greatly reduces the rule duplication and thus also the memory requirement, compared to HyperCuts. However, EffiCuts is known to provide slower packet classification speed than HyperCuts since there are many decision trees to look over for each packet.

## 3. Proposed Algorithm

In this section, we explain our partitioning algorithm that minimizes rule duplication. The proposed algorithm can improve both the memory requirement and the packet classification performance effectively by solving the limitation of existing decision tree algorithms. For an easy explanation of the proposed algorithm, we assume that rules consist of 5-tuple such as source IP, destination IP, source port, destination port, and protocol.

### 3.1. Motivation

In a decision tree algorithm, if we use more fields for partitioning a rule-set, the depth of the final decision tree is generally reduced, thereby improving the packet classification performance. However, this partitioning process does not use memory efficiently due to significantly generated redundant nodes. HyperCuts selects the partitioning field based on the number of unique matching ranges for each field in order to balance packet classification performance and memory usage. Unfortunately, this approach still has the following problems.

First, it does not consider wildcard fields in partitioning at all. HyperCuts internally adopts the equal-interval partitioning. That is, if the algorithm selects a field with a large number of unique matching ranges according to Equation (1) but it is unfortunately a wildcard field, many rules are duplicated during the partitioning process. Thus, in this case, it should consider wildcard fields and select a partitioning field to decrease the rule duplication and the memory requirement.

Second, HyperCuts does not determine the optimal number of partitions. If two partitioning fields lead to the same number of partitions, the number of partitions for a field with smaller matching range is reduced by half according to the internal algorithm. However, it can be better to choose a field with larger matching range in reducing the number of partitions. In conclusion, the performance of the final decision tree severely depends on the partitioning field selection algorithm and the number of partitions for the field. We can improve the ratio of memory requirement to packet classification performance by elaborately choosing the partitioning fields as well as the number of partitions.

### 3.2. Proposed Partitioning Algorithm

We now describe the proposed partitioning algorithm using wildcards in detail. Our algorithm consists of two sub-algorithms, the partitioning field selection algorithm and the partitioning number per field decision algorithm. We call the proposed algorithm Selection Based on Wildcards (SBW) for simplicity.

#### 3.2.1. Partitioning Field Selection Algorithm

We should determine the wildcard field for each node to be partitioned, in order to select the partitioning field minimizing the rule duplication. We recursively choose the partitioning node until the decision tree is completed, thereby considering the matching range of the current node. For example, let us assume that the current node has a partitioned matching range for Field 1 as (0,7), where the original full range of the field is (0,15). If we make partitions for the node based on Field 1 again, then we should consider the full matching range as (0,7) instead of (0,15).

**Figure 3:**
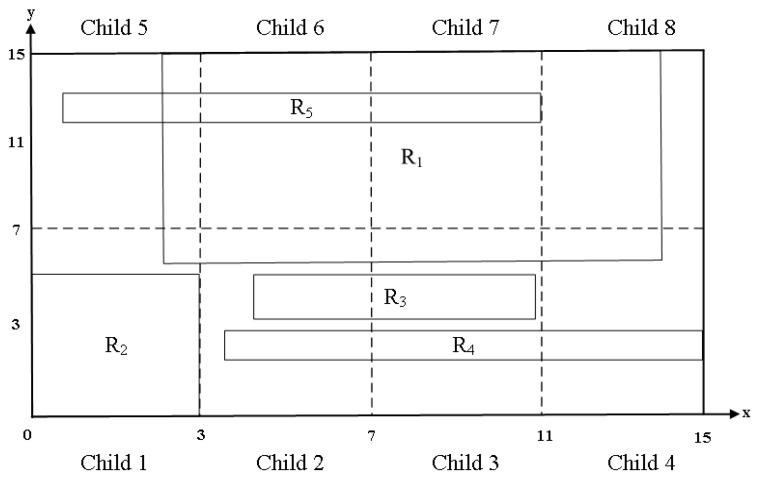
A rule-set of the root node represented in a two-dimensional plane.

However, Equation (4) of EffiCuts determines if a field is a wildcard or not using the full matching range of the field. For example, Figure 3 shows a two-dimensional representation of matching ranges for two fields in the rules stored in the root node. Note that Δ is set to 0.5. For rule $R_{1}$, we can compare the matching range of each field with the corresponding range of the field. Then, it is easy to see that all fields of $R_{1}$ are wildcards. On the other hand, for $R_{2}$, we can see that the matching ranges of all the fields occupy less than 0.5. Thus, $R_{2}$ does not have a wildcard field.

**Figure 4:**
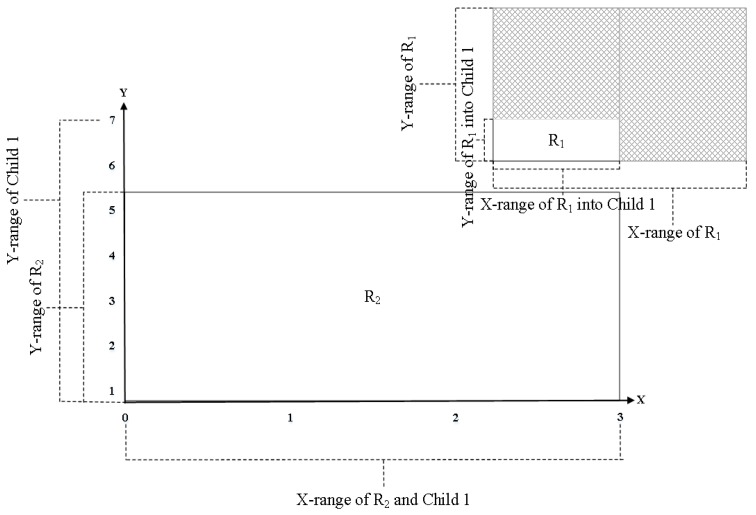
The sub-rule-set of Child 1 represented in a two-dimensional plane after partitioning the root node of Figure 3.

In the child node of the root node, the wildcard field of each rule can change. We consider Child 1, that is, one of eight child nodes created by partitioning the root node of Figure 3. Figure 4 shows a two-dimensional representation of the matching ranges for two fields in the rules stored in Child 1. For $R_{1}$ stored in Child 1, all fields occupy less than 0.5 of the corresponding full ranges, unlike at the root node. Therefore, $R_{1}$ stored in Child 1 has no wildcard field. On the other hand, all fields of $R_{2}$ occupy more than 0.5 when each field range is compared with its full range in Child 1. Therefore, all fields of $R_{2}$ in Child 1 are wildcard fields.

The original wildcard field is defined only for a root node, but we extend the concept of wildcard field to each child node also in a decision tree. To define the extended concept of wildcard, we denote all notations as follows:

- $N_{i}$: The ith node.
- $NK_{i,\min}^{k}$: Minimum value matching the k-th field at node $N_{i}$.
- $NK_{i,\max}^{k}$: Maximum value matching thek-th field at node $N_{i}.$
- $PK_{i,\min}^{k}$: Minimum value matching the k-th field of a given rule at node $N_{i}$.
- $PK_{i,\max}^{k}$: Maximum value matching the k-th field of a given rule at node $N_{i}$.

We now define the following condition for the k-th field of a rule stored in node $N_{i}$.

(5) $$\min\left\{PK_{i,\max}^{k},NK_{i,\max}^{k}\right\}-\max\left\{PK_{i,\min}^{k},NK_{i,\min}^{k}\right\}\geq \left(NK_{i,\max}^{k}-NK_{i,\min}^{k}\right)\cdot \Delta$$

Equation (5) considers the matching range for wildcard decision in the current node. However, it causes additional partitioning time since it calculates Equation (5) in every node during partitioning, until the final decision tree is completed.

The number of unique ranges is used as an indicator of whether rules are evenly distributed to child nodes or not. As the number increases, the rules tend to be uniformly distributed to the child nodes without significant rule duplication. For example, if there is only one range, the rules can be duplicated and distributed to all new child nodes. Then, the memory usage increases and the packet classification speed decreases.

A wildcard is also an indicator of the rule duplication degree. If there are many rules for which the selected partitioning field is a wildcard, it causes many rule duplications. It significantly increases the memory requirement. If such fields are frequently chosen as a partitioning field, the final decision tree will be huge. Therefore, the selected partitioning field should have rules without wildcards to minimize the rule duplication. With these observations, our proposed algorithm selects a field with a small number of wildcards and a large number of unique matching ranges as a partitioning field.

The proposed algorithm can be described as follows. First, the number of unique matching ranges and the number of wildcards are calculated for each field of every rule stored in the node. Then, the average values of the numbers for all fields are obtained. Finally, the partitioning preference coefficient is calculated by using these average values. The partitioning preference coefficient for the k-th field S(k) is defined as follows:

(6) $$S\left(k\right)=\{\begin{array}{c} \frac{u_{k}/\bar{u}}{{\left\{\left(\omega_{k}+1\right)/\left(\bar{\omega }+1\right)\right\}}^{2}},\;\;\;\;\;u_{k}\neq 1\\ \;\;\;\;\;\;\;\;\;\;0\;\;\;\;\;\;\;,\;\;\;\;\;\mathrm{otherwise} \end{array}$$

where $\omega_{k}$ is the number of wildcards for the k-th field and $\bar{\omega }=\frac{1}{d}\sum_{k=1}^{d}\omega_{k}$. In Equation (6), we use ($\omega_{k}+1)$ instead of $\omega_{k}$ to avoid dividing by zero. This coefficient represents how much the specific field is appropriate for partitioning. If the value is large, the field is suitable for the partitioning criterion.

Existing algorithms such as HyperCuts and EffiCuts select the partitioning field using the number of unique ranges as in Equation (1). However, the condition does not consider the number of wildcards at all, which causes considerable rule duplication problems. On the other hand, the proposed algorithm computes S(k) as in Equation (6) by considering the number of wildcards. Since it has no constraint such as $u_{k}\geq \bar{u}$ from Equation (1), all S(k) cannot be zero, simultaneously. It selects the two fields with the largest S(k) as partitioning fields through Equation (6). 

The existing algorithm such as HyperCuts or EffiCuts can theoretically choose multiple partitioning fields, even more than two, in contrast to ours. Partitioning using more fields generally shows the better partitioning result. However, we should note that in practice, the existing algorithm usually selects only two or fewer fields. This property guarantees that our algorithm does not achieve lower performance compared to the existing solutions although it just chooses two fields. When we calculate S(k) through Equation (6) for the rule-set in Table 1, we obtain the result of Table 2. In this example, Fields 2 and 3 are selected as partitioning fields.

#### 3.2.2. Partitioning Number per Field Decision Algorithm

After we choose two partitioning fields through the above-described algorithm, we should determine the number of partitions based on the selected fields. The existing algorithm, HyperCuts, initially determines the number of partitions for each field through Equation (2). After that, it determines the most efficient partitioning number by considering the combination of partitioning fields through Equation (3). If the numbers of partitioning fields are the same, the proposed algorithm tries to reduce the number of partitioning fields with a larger matching range as well as a field with a smaller matching range using Equation (3), in contrast to the existing algorithm. Then, we separately conduct partitioning for the two partitioning fields in advance on the current node, and select the most efficient number of partitions that minimizes rule duplication.

**Figure 5:**
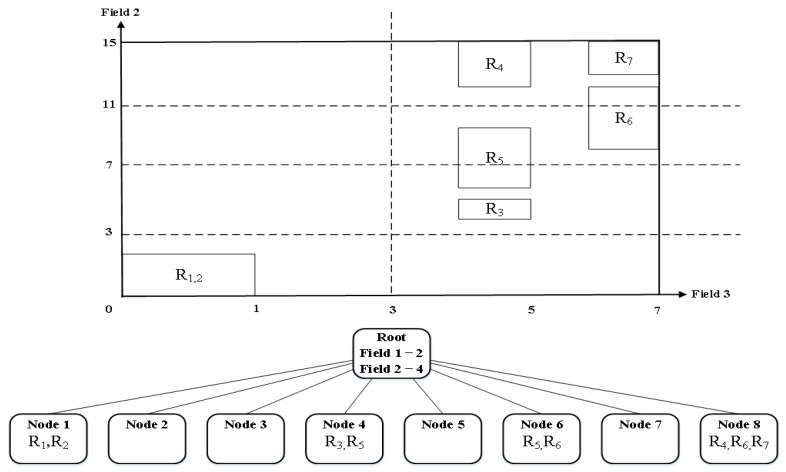
The generated final decision tree after decreasing the partitioning number of Field 3 from Table 2.

**Figure 6:**
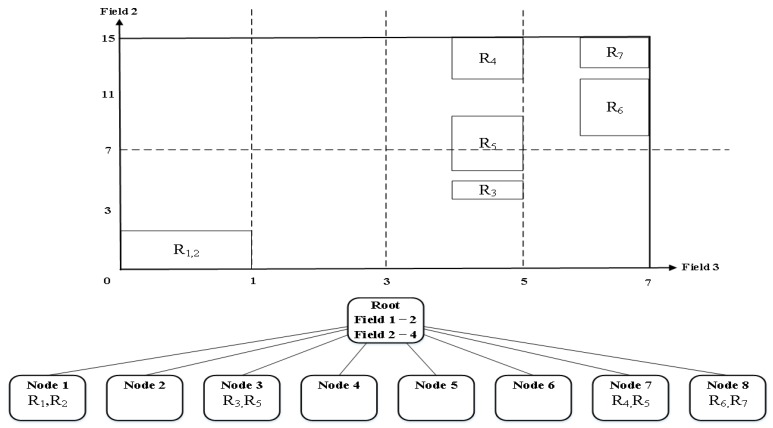
The generated final decision tree after decreasing the partitioning number of Field 2 from Table 2.

**Figure 7:**
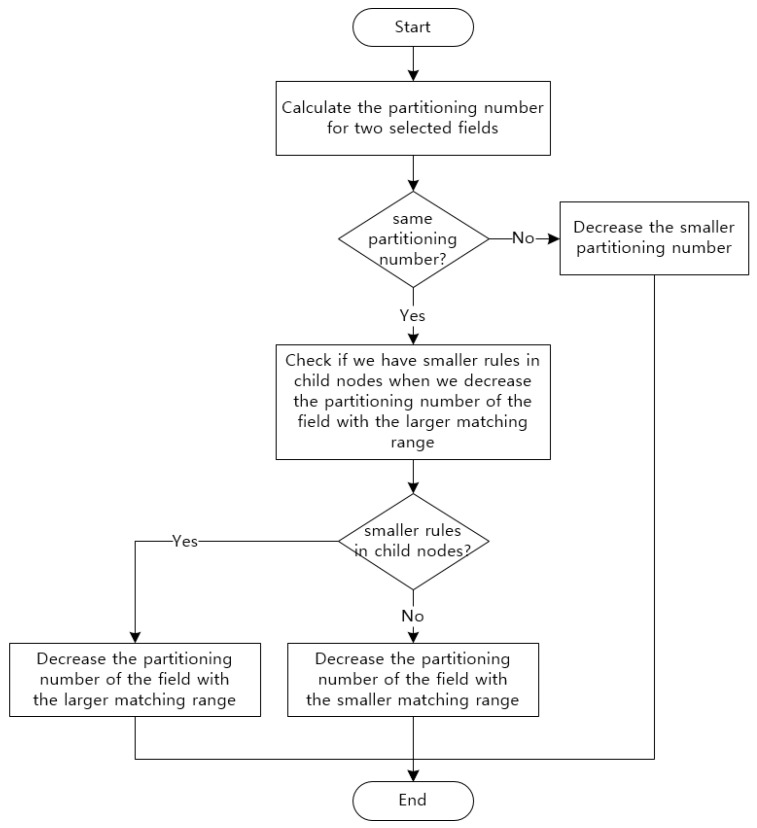
Proposed algorithm determining the optimal number of partitions.

Figure 5 shows the final decision tree generated by reducing the number of partitions of Field 3 among partitioning fields selected in Table 2 using Equation (3). In contrast, Figure 6 shows a final decision tree generated by reducing the number of partitions of Field 2. Let us assume that binth and space factor are set to 2. Figure 5 shows that it has smaller duplicated rules than that of Figure 1 which is generated by the existing algorithm. However, it still has more duplicate rules compared to Figure 6 although it generates a decision tree after reducing the number of partitions of Field 3. Therefore, we can know that it is necessary to reduce the number of partitions of the field having the largest matching range to perform a more efficient partitioning. Finally, we can make an algorithm that selects the partitioning number per field as shown in Figure 7.

### 3.3. Features of the Proposed Algorithm

Compared to HyperCuts and EffiCuts, our proposed algorithm has unique features such as high flexibility, low memory requirement for building and maintaining decision trees, fast building speed, and fast classification performance.

#### 3.3.1. High Flexibility

The proposed algorithm adopts a new partitioning field selection algorithm that minimizes the memory requirement in the partitioning process of HyperCuts. Therefore, it can be easily applied to partitioning process of all packet classification algorithms based on HyperCuts. For example, EffiCuts classifies a rule-set into sub-rule-sets according to the number of wildcard fields and then partitions each sub-rule-set in the same way as HyperCuts. Therefore, the proposed algorithm can be combined into the partitioning process of EffiCuts. In this case, although EffiCuts can reduce memory requirement significantly compared to HyperCuts, our algorithm can reduce the memory requirement more than EffiCuts.

#### 3.3.2. Low Memory Requirement

As mentioned earlier, the existing algorithm does not consider wildcards at all in selecting partitioning fields. However, rule duplication is a serious problem caused by wildcard field and the memory requirement increases proportionally to the number of wildcard fields.

The proposed algorithm considers both the number of unique matching ranges and the number of wildcards to reduce the memory requirement. It chooses fields with the smallest wildcard number as partitioning field by assigning more weight to the number of wildcards than the number of the unique matching ranges. This minimizes the rule duplication for each partitioning process, which can significantly reduce the decision tree size and the memory requirement for building the tree.

#### 3.3.3. Fast Decision Tree Building Speed

One of the goals of the proposed algorithm is also to build the decision tree in a short time. To generate a decision tree fast, the partitioning algorithm should minimize the size of rules in the generated child nodes for each partitioning. The proposed algorithm finds the most efficient partitioning number by partitioning the given rule-set in advance according to the obtained partitioning number. Such an approach can increase the partitioning time more than the original partitioning algorithm. However, it can perform more efficient partitioning, therefore, generating child nodes with very small number of rules. Although it takes a longer time in the partitioning process, the total time for generating the final decision tree can be reduced.

#### 3.3.4. Improved Classification Performance due to Memory Reduction

The decision tree-based packet classification algorithm can easily trade off the memory requirement and the packet classification performance using “binth” and “space factor” parameters. Accordingly, if the memory requirement is increased, the classification performance can be improved by adjusting the parameters. The proposed approach greatly reduces the memory requirement, i.e., just 1/5 compared to EffiCuts, while maintaining the classification performance almost the same as the original algorithm. It means that the packet classification performance can be further improved by increasing the memory requirement until it reaches to the original requirement size through adjusting the parameters.

## 4. Performance Evaluation

Our proposed algorithm can be used with various classification algorithms based on decision tree to improve scalability in terms of supportable rule-set size. In this section, we apply our algorithm to HyperCuts and EffiCuts, which are well known for high classification performance and high scalability, and compare the performances before and after applying our algorithm. Since HyperCuts is one of the fastest classifications and EffiCuts is one of the most memory efficient classification algorithms among decision tree-based ones, we choose them as target algorithms to be applied with our SBW. For performance evaluation, we use three rule-sets, i.e. Access Control List (ACL), Firewall (FW), and IP Chain (IPC), generated by ClassBench [18]. For each type of rule-set, the rule-set size is set to 20K, 40K, 60K, 80K, and 100K, respectively. For all cases, we set binth to 16 and space factor to 4 that are typically optimal values for existing algorithms. We adopt the following three metrics for performance evaluation.

• Memory requirement per rule

It is the memory space required for a single rule. This metric is important for scalability of a packet classification algorithm with the rule-set size. These days, the rule-set size installed in a network equipment is increasing exponentially. Scalability is one of the most significant features of the packet classification algorithms. The required memory size per rule should be constant or decrease as the rule-set size increases, to support large-scale rule-sets.

• Packet classification performance

We measure the packet classification performance by the memory size accessed for packet classification procedure, because it is a bottleneck of the classification performance. The classification algorithm extracts key values from the head of each packet, and recursively searches the appropriate child node by comparing the keys with the matching condition of the traversing node in a decision tree. The search procedure stops when it reaches a leaf node. Then, the algorithm compares the keys with each rule stored in the leaf node. We measure the accessed memory size in bytes to accurately estimating the performance. For precise performance evaluation and analysis, we measure both the average and the worst performances. Average classification performance shows the overall performance of network equipment. On the other hand, the worst-case performance determines the specification of network equipment. 

• Table building time

Table building time refers to the time required to generate the final decision tree. We rebuild the decision tree whenever a rule changes. Even when the rule changes frequently, the packet classification algorithm should build the search table fast enough to satisfy the requirements of services such as V2X.

### 4.1. Memory Requirement per Rule

**Figure 8:**
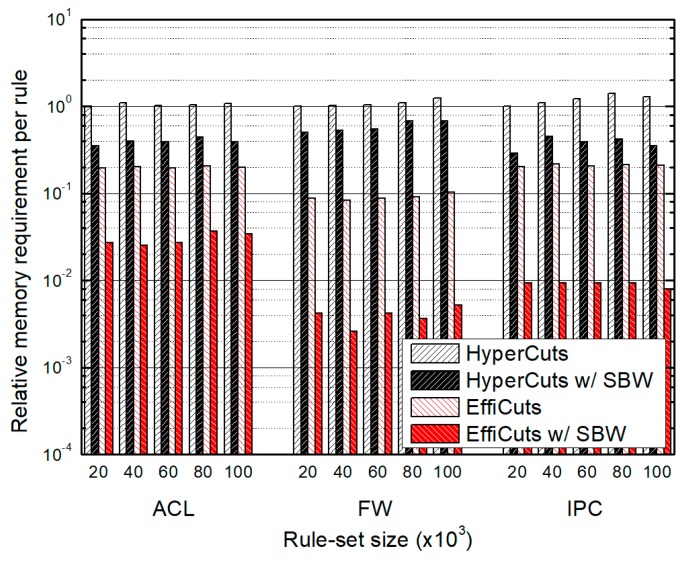
The relative memory requirement per rule according to rule-set type and size, which are normalized by those of HyperCuts, respectively.

Figure 8 illustrates the average memory requirement of each scheme for one classification rule. For easy comparison, we normalize the memory requirements of ACL, FW, and IPC rule-sets by those of HyperCuts, respectively. We can see that our algorithm greatly reduces the required memory size compared to HyperCuts and EffiCuts. EffiCuts requires smaller memory than HyperCuts. However, when our SBW is combined with EffiCuts, the benefit is much outstanding. The average reduction ratio of memory is 60% for HyperCuts with SBW but 89% for EffiCuts with SBW. The memory size is critical for scalability, so we conclude that our algorithm is capable of supporting large rule-sets.

### 4.2. Packet Classification Performance

**Figure 9:**
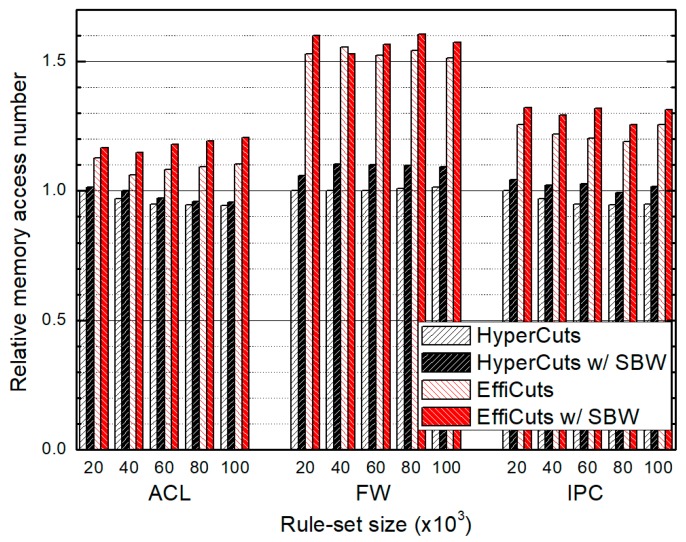
The relative average memory access number for classifying the entire rule-set according to rule-set type and size, which are normalized by those of HyperCuts, respectively.

**Figure 10:**
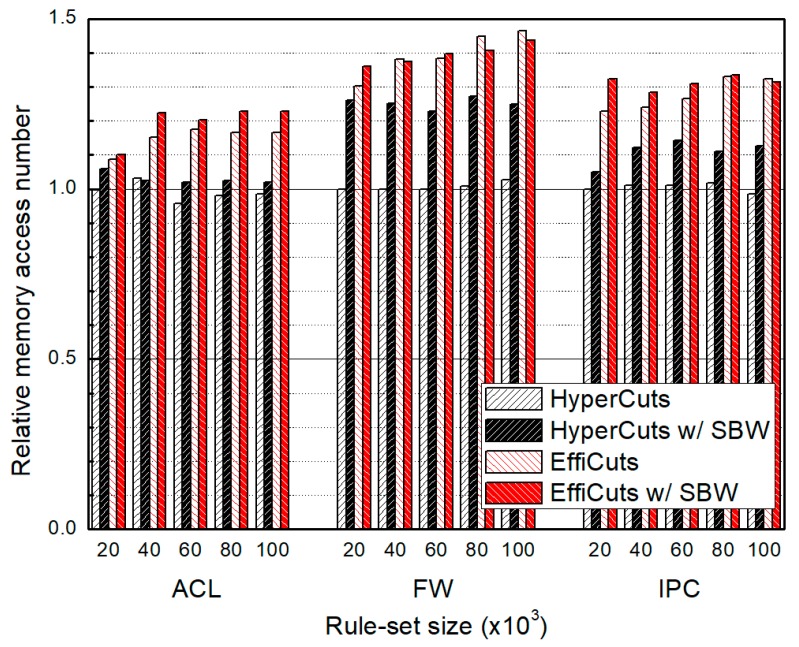
The relative worst memory access number for classifying the entire rule-set according to rule-set type and size, which are normalized by those of HyperCuts, respectively.

Figure 9 and Figure 10 show the relative average and the relative worst classification performance, respectively. The proposed algorithm avoids rule duplication by choosing the partitioning fields in terms of memory efficiency rather than classification performance. Thus, the classification performance of our algorithm is inevitably degraded. However, the increasing ratios for average classification performance are just 7% for HyperCuts with SBW and 5% for EffiCuts with SBW. The ratios for the worst-case performance are 13% for HyperCuts with SBW and 2.5% for EffiCuts with SBW. We note that the performance degradation is almost equal regardless of the rule-set size, which is very important for scalability.

### 4.3. Table Building Time

**Figure 11:**
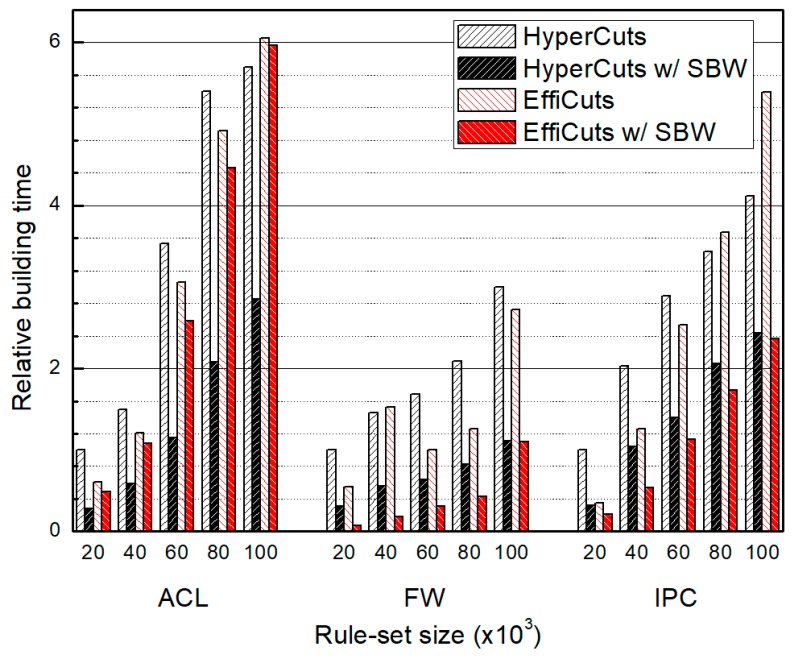
The relative building time of packet classification tree according to rule-set type and size, which are normalized by those of HyperCuts, respectively.

Figure 11 compares decision tree building times of each algorithm. Our proposed algorithm finds the optimal field by partitioning the rule-set according to all the selected fields and choosing the field with the minimal duplication rules to achieve near optimal partitioning. Therefore, this approach takes a relatively long time. As a return, the rule duplication is minimized and therefore, the average number of rules in the generated child nodes reduces. So, the number of nodes to be partitioned in the process can be also reduced, which decreases the table building time significantly. Since the number of child nodes is more sensitive to the building time than repetitive partitioning selection, our decision tree is faster than the competitors are.

Since SBW focuses on minimizing duplicated rules, HyperCuts or EffiCuts with SBW can be improved significantly in terms of memory requirement and building time, essential metrics to support V2X services in SDNs. Although the trade-off relation between classification speed and memory requirement exists, we can see that our approach can effectively minimize the side-effects of SBW, so we can have only marginally decreased classifciation performance. From the experiments, we confirm that we can obtain many advantages using SBW for existing algorithms.

## 5. Conclusions

In this paper, we have proposed a new partitioning algorithm to resolve the high memory requirement and low scalability for existing packet classification algorithms. Differently from partitioning algorithms of the decision tree-based classification algorithms, we exploit not only the number of unique range elements but also the number of wildcard fields in the rule partitioning. Our algorithm selects a partitioning field that minimizes the rule duplication, by weighting the number of wildcard fields more than the number of unique ranges. For the field chosen, we determine the optimal number of partitions at the current node. Repeating this procedure, we construct a decision tree consuming the minimal memory space. We observe that existing packet classification algorithms with our algorithm outperforms them without ours in various scenarios. In some cases, search time can increase slightly but with significantly reduced memory usage. When we use the same amount of memory by adjusting the parameters such as “binth” and “space factor”, search performance improves highly. Therefore, we conclude that the proposed scheme attains the high-speed packet classification while supporting a large size rule-set simultaneously.

## Figures and Tables

**Table 1:** Example of a rule-set where the field sizes of Fields 1 and 2 are 4-bit, and those of Fields 3 and 4 are 3-bit, respectively.

Rule	Field 1	Field 2	Field 3	Field 4
Rule 1	(0,8)	(0,1)	(0,2)	(0,5)
Rule 2	(6,9)	(0,1)	(0,2)	(0,5)
Rule 3	(0,15)	(4,5)	(4,5)	(0,5)
Rule 4	(9,10)	(12,15)	(4,5)	(6,7)
Rule 5	(6,15)	(6,10)	(4,5)	(6,7)
Rule 6	(0,8)	(8,12)	(6,7)	(6,7)
Rule 7	(0,2)	(13,15)	(6,7)	(6,7)

**Table 2:** Partitioning field selection by calculating S(k) from Table 1.

	Field 1	Field 2	Field 3	Field 4
Number of unique matching ranges	6	6	3	2
Average number of unique matching ranges	4.5			
Number of wildcards	4	0	0	0
Average number of wildcards	1.75			
S(k)	0.19	4.08	2.04	0.14
Selected partitioning fields	Field 2, Field 3			
